# Utilizing Magnetic Levitation to Detect Lung Cancer-Associated Exosomes

**DOI:** 10.1021/acssensors.4c00011

**Published:** 2024-03-23

**Authors:** Alper Baran Sözmen, Ahu Arslan-Yildiz

**Affiliations:** Bioengineering Department, Izmir Institute of Technology, 35430 Izmir, Turkey

**Keywords:** liquid biopsy, lung cancer, magnetic levitation, exosome, exosomal membrane protein detection

## Abstract

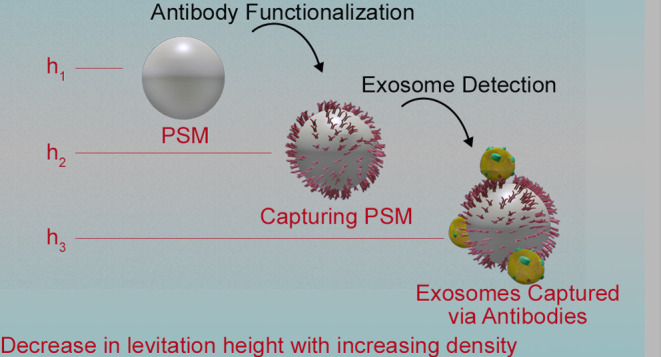

Extracellular vesicles, especially exosomes, have attracted attention in the last few decades as novel cancer biomarkers. Exosomal membrane proteins provide easy-to-reach targets and can be utilized as information sources of their parent cells. In this study, a MagLev-based, highly sensitive, and versatile biosensor platform for detecting minor differences in the density of suspended objects is proposed for exosome detection. The developed platform utilizes antibody-functionalized microspheres to capture exosomal membrane proteins (ExoMPs) EpCAM, CD81, and CD151 as markers for cancerous exosomes, exosomes, and non-small cell lung cancer (NSCLC)-derived exosomes, respectively. Initially, the platform was utilized for protein detection and quantification by targeting solubilized ExoMPs, and a dynamic range of 1–100 nM, with LoD values of 1.324, 0.638, and 0.722 nM for EpCAM, CD81, and CD151, were observed, respectively. Then, the sensor platform was tested using exosome isolates derived from NSCLC cell line A549 and MRC5 healthy lung fibroblast cell line. It was shown that the sensor platform is able to detect and differentiate exosomal biomarkers derived from cancerous and non-cancerous cell lines. Overall, this innovative, simple, and rapid method shows great potential for the early diagnosis of lung cancer through exosomal biomarker detection.

Cancer has become one of the leading causes of death in the world, and it is one of the diseases with the highest incidence, especially in developed countries.^1^ Despite the high mortality rate, 40% of cancer cases are curable when diagnosed at an early stage.^2,3^ However, early diagnosis is difficult due to the exclusion of cancer screening from standard check-up procedures. This exclusion is based on the highly invasive nature of tissue biopsy, which is the current gold standard in cancer diagnosis. Moreover, limited access to healthcare, inadequate diagnostic tools, and lack of symptoms in the early stages also hinder the early diagnosis of the disease.^4−6^ Hence, in the context of early diagnosis, tissue biopsy is not considered as an effective method.

Recent advancements in technology have led to development of non-invasive methods for cancer detection, like liquid biopsy.^7,8^ Liquid biopsy is a diagnostic method that involves detecting circulating tumor DNA (ctDNA), circulating tumor cells (CTCs) or extracellular vesicles (EVs) in bodily fluids, such as blood or urine. Exosomes are a class of EVs that are intercellular signaling organelles with multiple functions, playing a role in communication, reflecting the cell’s physiological status, and having potential applications in diagnostics.^9^ Cancer cell-derived exosomes can be utilized to trace the origin, stage, and location of the disease and may hold the potential to become distinctive cancer biomarkers. Current studies mostly focus on the genetic material that is carried by exosomes. On the other hand, exosomes carry information not only in the form of genetic material but also in their protein content. Therefore, membrane proteins of exosomes (ExoMPs) are easy-to-reach potential biomarkers.^10^ Tetraspanins such as CD9, CD36, CD44, CD63, CD81, and CD151 are membrane proteins found in exosomes and are investigated as potential biomarkers for various types of cancer. However, due to the requirement of expertise, developed facilities, or both, current ExoMP detection methods such as ELISA, western blot, and EV arrays that investigate cancer diagnosis are quite demanding.^11−16^

Magnetic levitation (MagLev) is a new detection methodology in the biosensor field, which utilizes magnetic force to balance gravitational force and provide a density-based detection technique.^17−20,26−30^ MagLev-based sensor platforms were reported for the analysis of biological and non-biological substances. MagLev-based sensor platforms are sensitive enough for protein assays, and they offer advantages such as adaptability, simplicity, and no need for sophisticated instruments.^21−30^ In our previous studies, optimization of a MagLev-based methodology was carried out and the method demonstrated a lower protein detection limit of 4.1 ng/mL, which highlights the potential of it for various biosensing applications.^31^ In conclusion, MagLev-based sensors have the potential to be used in liquid biopsy, thus providing a new frontier for innovative methodologies for cancer diagnosis.

In this study, a MagLev-based sensor platform for detecting exosomes through targeting ExoMPs is proposed. Three ExoMPs, CD81, EpCAM, and CD151, were chosen as target biomarkers to capture exosomes, cancerous exosomes, and non-small cell lung cancer (NSCLC)-derived exosomes, respectively.^13,14^ Polystyrene microspheres (PSMs), which are functionalized with respective antibodies of the aforementioned proteins, were utilized as capturing agents. The MagLev heights of PSMs were measured before and after capturing proteins, and quantification was carried out via image analyses. Then, the A549 NSCLC cell line, which releases exosomes that contain CD81, EpCAM, and CD151, was used as a model cell line for *in vitro* exosome detection. MRC5 healthy lung fibroblast-derived exosomes were used as control groups throughout the study. Overall, the MagLev platform was utilized for the first time to detect exosomes, and it allowed simple, sensitive, accurate, and rapid density-based detection that could have significant implications in liquid biopsy applications.

## Materials and Methods

### Materials

In this study, following materials were used: poly(methyl methacrylate) (PMMA) sheets (NetPlexi, Turkey); mirrors; N52-grade neodymium (NdFeB) magnets (K&J Magnetics); a glass capillary channel (VitroCom); carboxylated polystyrene microspheres (PSM) of 20 μm diameter (Lab261, PST20KC); phosphate buffer saline (PBS, GIBCO); paramagnetic solution (Gadobutrol, Bayer); 1-ethyl-3-(3-(dimethylamino)propyl)carbodiimide hydrochloride (EDC, Sigma-Aldrich, E7750); *N*-hydroxysuccinimide (NHS, Sigma-Aldrich, 8045180025); EpCAM (Abcam, ab155637), CD81 (Abcam, ab226886), and CD151 (Abcam, ab152269) proteins and their respective antibodies anti-EpCAM (Abcam, ab71916), anti-CD81 (Abcam, ab109201), and anti-CD151 (Abcam, ab185684); cell lines A549 (ATCC, CCL-185) and MRC5 (ATCC, CCL-171); DMEM (GIBCO, Thermo Fischer Scientific); FBS (GIBCO, Thermo Fischer Scientific); exosome isolation kit (Norgen Cell Supernatant Exosome Isolation Midi Kit); paraformaldehyde (PFA); and rhodamine-conjugated anti-rabbit antibody (ABclonal, A5040).

### MagLev Sensor Platform

The MagLev sensor platform was fabricated as described elsewhere.^24,31,32^ Briefly, a 2 mm PMMA frame was fabricated through laser ablation via a Versa Laser VLS 2.30 (Universal Laser). The frame included saddles for four mirrors, two magnets, and a glass capillary channel. Two NdFeB magnets were arranged in an anti-Helmholtz configuration, and four mirrors provided an image of the capillary’s lateral section. A Zeiss Axio Observer microscope captured images of the samples introduced through a borosilicate capillary channel.

### Optimization of Microsphere Functionalization

PSMs were utilized as capturing agents of proteins as described elsewhere.^31,32^ For this purpose, PSMs were functionalized with the respective antibodies of target proteins (EpCAM, CD81, and CD151). Antibody (Ab) concentrations of 0.05, 0.25, 0.5, 1, 2.5, 25, and 50 μg/mL were used to investigate the surface saturation of PSMs. Functionalization was carried out using the EDC/NHS protocol.^30^ For this purpose, 0.4 mM EDC and 0.1 mM NHS were added to the PSM suspension, and surface activation was carried out. Then, Ab solution was introduced into the mixture and incubated for an hour. Afterwards, excess Ab, EDC, and NHS were removed via centrifugation of PSMs, and functionalized PSMs were resuspended in 50 mM Gx for MagLev height measurement. The suspension was introduced into the capillary channel in the MagLev platform, and after 10 min, PSMs reached an equilibrium levitation height. Levitated PSMs were visualized using light microscopy, and the levitation heights of PSMs were measured via MATLAB R2018b.

### Solubilized Protein Detection with the MagLev Sensor Platform

The MagLev sensor platform was used for the detection of solubilized EpCAM, CD81, and CD151 proteins separately.^30^ Functionalized PSMs were resuspended in PBS (pH 7.0) and mixed with protein solutions with concentrations of 1, 5, 10, 50, and 100 nM equivolumetrically. After 1 h of incubation, excess protein was removed via centrifugation, and PSMs were resuspended in 50 mM Gx. Lastly, MagLev measurements were carried out, and related calibration curves were prepared using levitation height differences. All data were subjected to normality tests and ANOVA.

### Cell Culture, Exosome Isolation, and Characterization

A549 NSCLC cell line as the experimental group and MRC5 healthy lung fibroblast cell line as the control group were cultured in DMEM and supplemented with 10% FBS (fetal bovine serum) at 5% CO_2_ and 37 °C. Cell culture supernatants (CCS) were used as exosome sources for the remainder of the study. Exosomes were isolated utilizing Norgen’s Cell Supernatant Exosome Isolation Midi Kit, and exosome isolation was realized as described in the isolation kit manual.

#### Exosome Size Analyses

A NanoPlus DLS Nano Particle Size and Zeta Potential Analyzer (DLS—Particulate Systems) was used to determine the sizes of isolated exosomes. Measurements were carried out in triplicate for various dilutions of exosome isolates.

#### Exosome Number Calculation

A theoretical calculation was carried out for exosomes utilizing the equations that have been given in the literature.^33−34,35^ First, standard liposomes with 100 nm diameter were prepared using phosphatidylcholine (PC) via extrusion. Then, DLS measurements were carried out for these liposomes with several dilution rates, and respective liposome numbers were calculated theoretically (1.14 × 10^11^ liposomes/mL with no dilution and 3.5 × 10^7^ liposomes/mL at the highest dilution). Size measurement result peaks of liposomes and *in vitro* exosomes were utilized to deduce a proportional relation between them, and this relation was utilized to calculate the exosome numbers.

#### Immunostaining of ExoMPs

Fixation of isolated exosomes was carried out using 4% PFA, and a routine immunostaining procedure was applied separately with the following antibodies: anti-EpCAM, anti-CD81, and anti-CD151. Rhodamine-conjugated anti-rabbit antibodies were used to stain exosomes, and then images were captured through fluorescence microscopy (Zeiss Axio Observer).

### Exosome Detection with the MagLev Sensor Platform

Exosome detection was carried out by the MagLev platform; isolates from cell lines A549 and MRC5 were used for this purpose. Either anti-EpCAM-, anti-CD81-, or anti-CD151-functionalized PSMs were used to capture membrane protein-containing exosomes. Isolated exosomes were first diluted (1:2), and than the sensor platform was used to detect the exosomes. Each of the experimentation sets was carried out in triplicates. After capturing exosomes, PSMs reached an equilibrium height in 10 min in the capillary, and then, they were visualized through light microscopy, and MagLev heights of PSMs were measured via MATLAB 2018.^32^ MagLev height differences were subjected to normality tests and ANOVA.

## Results and Discussion

**Figure 1:**
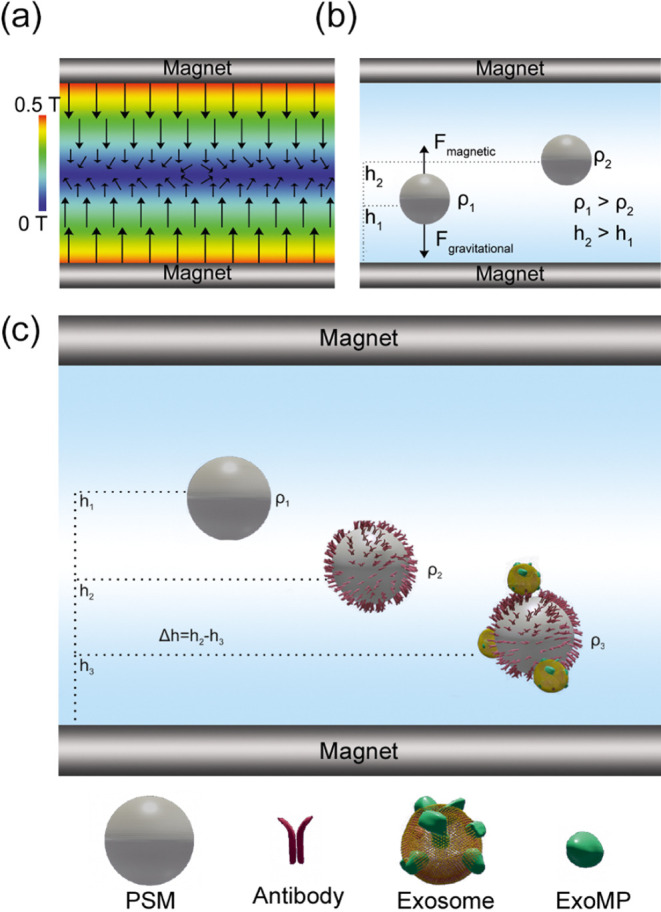
Detection principle schematic of the MagLev sensor. (a) Simulation of magnetic force distribution through the capillary of the MagLev sensor platform provided by two NdFeB magnets in anti-Helmholtz configuration. (b) Magnetic levitation of PS microspheres in the gravitation-free environment and their levitation heights with specific densities ρ_1_ and ρ_2_ in the sensor platform. (c) Magnetic levitation height variation during antibody functionalization of PSMs and exosome capturing, where ρ_1_ < ρ_2_ < ρ_3_.

The working principle of the MagLev sensor platform is based on measurement of the relative densities of the particles. In order to mimic an environment without gravitational effects, a magnetic field is applied by utilizing magnets. At a certain levitation height, gravitational force and magnetic force are applied onto a particle to equalize it; hence, the particle levitates at that certain height. This height is solely dependent on the density of the particle. Herein, PSM levitation heights (*h*) change due to the changes in their density, which occurs when they are functionalized with antibodies and further interact with antigens. PSMs have a density of 1.06 g/mL when they are functionalized with capturing antibodies (Ab), that leads to an increase in their density. Interaction of Ab-functionalized PSMs with ExoMPs or exosomes causes a further increase in their density. MagLev height decreases (*h*_1_ > *h*_2_ > *h*_3_) as the density of the PSM–protein complex increases (ρ_1_ < ρ_2_ < ρ_3_), as depicted in Figure 1. Here, the MagLev height difference (Δ*h*) is utilized to establish a relation between Δ*h* and analyte concentration.

### Optimization of Microsphere Functionalization

Ab concentration optimization was carried out via functionalization of PSMs with varying concentrations (0.05, 0.25, 0.5, 1, 2.5, 25, and 50 μg/mL) of anti-EpCAM. After functionalization, PSMs were introduced into the Maglev platform, and they were visualized via a light microscope.

**Figure 2:**
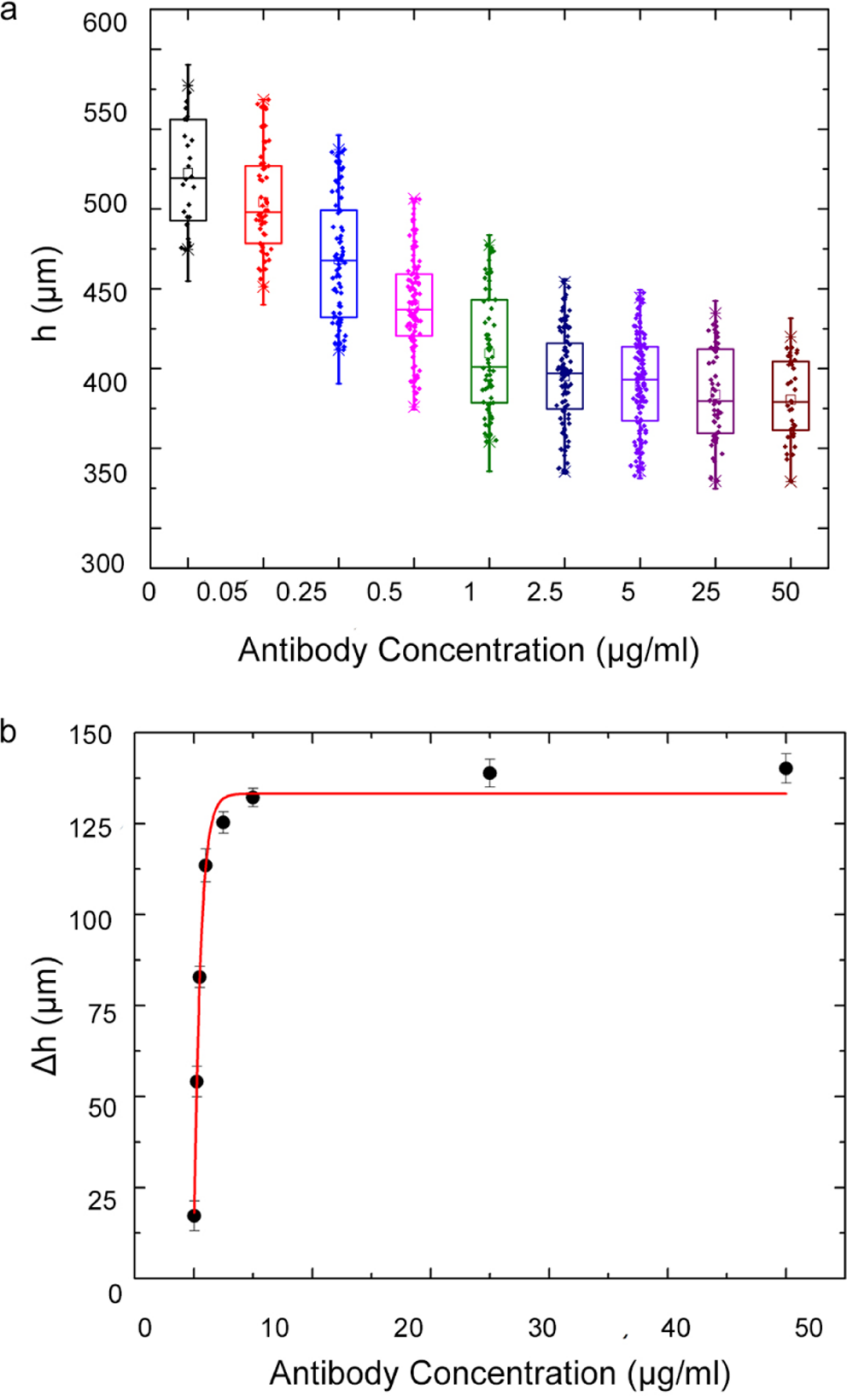
Surface saturation of PSMs with the antibody. (a) Distribution of PSM MagLev heights with increasing antibody concentration. (b) Calibration curve of PSM MagLev height differences with standard error, functionalized with various concentrations of the antibody (*N* = 60).

Following the process, levitation heights were measured via MATLAB, and the results are summarized in Figure 2. As expected, an increase in Ab concentration led to a decrease in levitation height due to the increase in PSM–Ab complex density. This height decrease confirmed the surface functionalization with Ab.

Figure 2a indicates that PSM surfaces get saturated to Ab between 5 and 50 μg/mL antibody concentrations, where levitation height stops decreasing with increasing Ab concentration. Non-linear regression analyses were also carried out to confirm this claim, and Figure 2b shows the regression plot. The results in Figure 2b further supported that 25 μg/mL Ab concentration is sufficient to saturate the PSM surface. The descriptive statistical analyses and normality test results showed that the data were significantly drawn from a normally distributed population (Table S1). Thereafter, one-way ANOVA was performed [*F*(8.698) = 154.1, *p* < 0.0001], and contrast tests showed that 5 μg/mL was the sufficient concentration for PSM surface saturation. Also, there was no significant levitation height difference between 5, 25, and 50 μg/mL (*p* = >0.999); therefore, 25 μg/mL Ab concentration was utilized to guarantee complete surface saturation for the remainder of this study.

### Solubilized Protein Detection with the MagLev Sensor Platform

**Figure 3:**
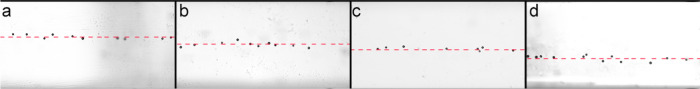
Light microscopy images acquired during EpCAM detection; dashed red lines indicate the mean levitation height of the related PSMs. (a) bare PSMs, (b) PSMs with anti-EpCAM, (c) PSMs with anti-EpCAM + 5 nM EpCAM, and (d) PSMs with anti-EpCAM + 100 nM EPCAM.

The MagLev sensor platform was tested with solubilized standard proteins of EpCAM, CD81, and CD151 to calculate detection limits. PSMs were functionalized with 25 μg/mL Ab and incubated in corresponding protein solutions before image capturing. Captured images were utilized to calculate the *h* and relevant Δ*h* values for further investigation. Figure 3 ensamples the light microscopy images of PSMs through the detection of solubilized EpCAM standard.

**Figure 4:**
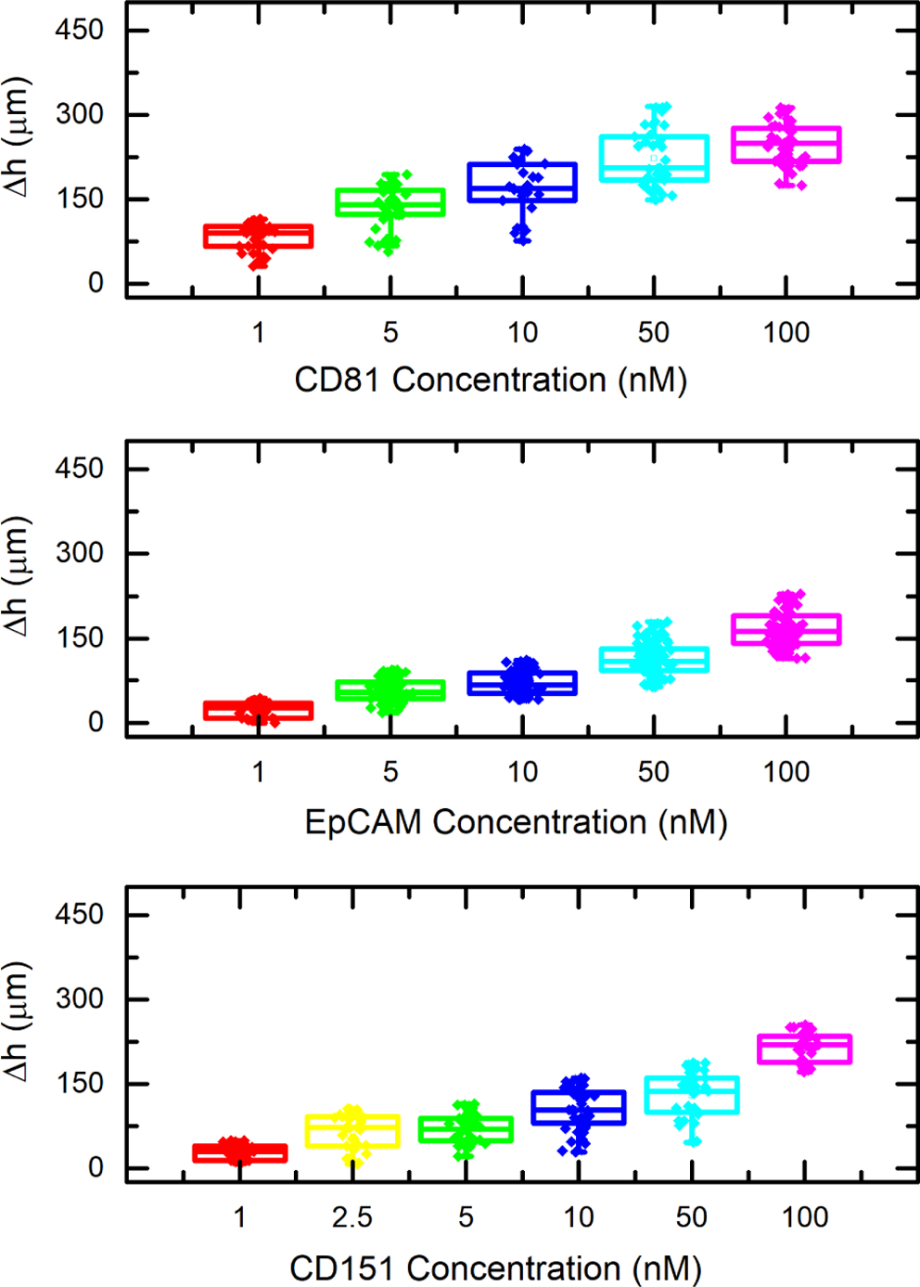
Protein detection via the MagLev sensor platform; results acquired for each solubilized protein standard (*N* = 40).

The results for each protein are listed in Figure 4. As the figure describes, all three proteins, EpCAM, CD81, and CD151, can be detected in the studied range. Descriptive statistical analyses, Kolmogorov–Smirnov normality test, and linear fitting were then applied. All results showed normal distribution for all replications for each protein and concentration (*p* > 0.05).

The Δ*h* difference between EpCAM and the other two proteins can be explained by the density difference of proteins. As discussed in the literature, average protein density is a molecular weight (MW)-based property, especially for proteins with a MW lower than 40 kDa.^35^ The density slowly increases after that limit and shifts from 1.35 to 1.52 g/mL; where EpCAM, CD81, and CD151 proteins reside in between, and this is the cause of Δ*h* difference between solubilized proteins, as shown in Table 1.

**Table 1:** Mean Δ*h* Values When the Platform is Utilized to Detect Solubilized Standard Proteins EpCAM, CD81, and CD151 with Various Concentrations^a^

	mean Δ*h* (μm)		
protein concentration (nM)	EpCAM (ρ ∼ 1.40 mg/cm^3^)	CD81 (ρ ∼ 1.46 mg/cm^3^)	CD151 (ρ ∼ 1.44 mg/cm^3^)
1	N/A^b^	82.669	22.668
5	57.530	136.840	68.945
10	71.0520	167.907	102.313
50	114.694	222.625	129.610
100	167.447	247.216	206.235

a Density values of ExoMPs are calculated as described by Craievich et al.^35^

b No significant difference.

Overall, Ab–protein interactions for EpCAM, CD81, and CD151 were successfully visualized, and statistical analysis results demonstrated that each experimentation group showed normal distribution (*p* > 0.05). Acquired data were used for both limit of detection (LoD) and limit of quantification (LoQ) determination; for this purpose, a calibration curve was fitted for each protein, and the calculated values are given in Table 2 for each protein.

**Table 2:** Detection and Quantification Limits for EpCAM, CD81, and CD151 Proteins and Related Linear Correlation Results

protein	LoD (nM)	LoQ (nM)
EpCAM	1.324	3.972
CD81	0.638	1.915
CD151	0.722	2.165

As previously mentioned, the density of a protein decreases with increasing MW, and the differences between Δ*h* values of studied proteins can be explained by these density variations. Moreover, these variations result in a difference in LoD and LoQ; for example, CD81 has the lowest MW of 26 kDa,^36^ which also corresponds to the lowest LoD and LoQ. On the other hand, CD151 has an MW of 29 kDa, and EpCAM has an MW of 40 kDa,^37^ which leads to higher LoD and LoQ values than the other two proteins. After the assessment of the sensor platform with protein standards, the study moved on to testing with *in vitro* exosome samples.

## Exosome Characterization

### Size and Concentration of Exosomes

Cell lines A549 and MRC5 were cultured, and exosomes were isolated via an isolation kit. Prior to sensor platform testing, the characterization of isolated exosomes was carried out. Exosome size and polydispersity index (PdI) were measured via DLS, and related distribution peak areas were used for the calculation of the exosome number in each isolate. Table 3 shows results obtained via DLS measurements and consecutive calculations.

**Table 3:** Exosome Diameter and Exosome Concentration for the Exosome Isolates

sample	exosome per mL (in isolate)	mean diameter (nm)
A549 cell line	2.90 × 10^9^	107.7
MRC5 cell line	1.80 × 10^9^	84.5

A549 isolates contained a higher number of exosomes compared to the control cell line, which was expected based on the literature. Studies showed that cancerous cell lines generate a higher number of exosomes compared to healthy ones, which supports our findings in this study.^38^ Moreover, their size also proved to be in between 30–150 nm, which is the recorded range for exosomes.^39^

### Immunostaining of ExoMPs

**Figure 5:**
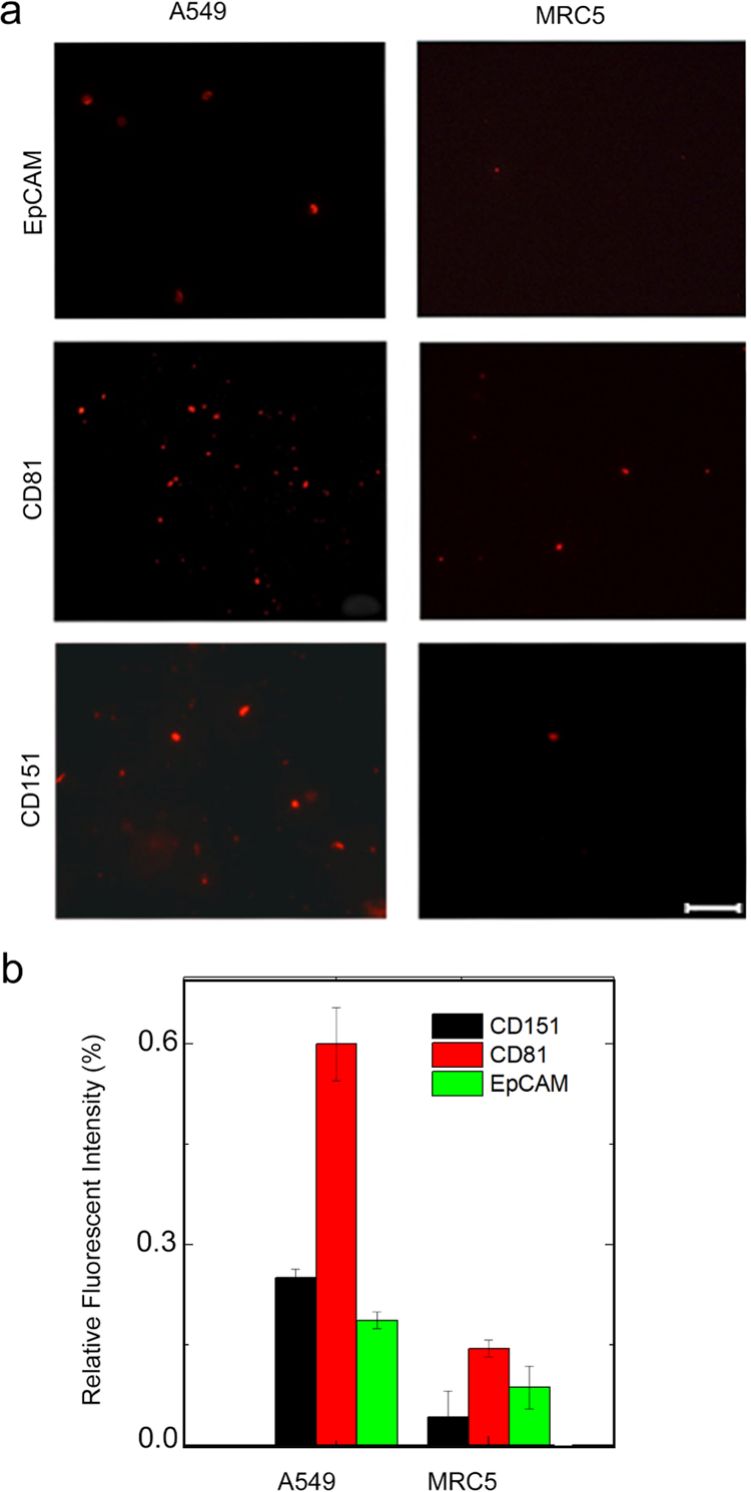
Immunostaining study for each of the target proteins applied onto exosome isolates from studied cell lines. (a) Microscopy images (scale bar: 50 μm) and (b) relative fluorescent intensity results.

Immunostaining of exosomes that are isolated from A549 and MRC5 cell lines was carried out for further characterization. ExoMP (EpCAM, CD81, and CD151)-containing exosomes were targeted and stained *in-situ*. Images acquired by fluorescence microscopy can be seen in Figure 5a for each protein and each *in-vitro* exosome sample, and related fluorescence intensity levels are given in Figure 5b. As shown in the figure, exosomes originating from the A549 cell line had much higher expression rates of CD151. Additionally, a higher amount of CD81 signal suggests a higher level of exosome generation. Literature suggests that exosome samples gathered from patients with NSCLC have elevated levels of CD151 in their content. Moreover, such studies have shown a higher number of exosomes in patient samples compared to healthy ones.^13,14^

As expected, the A549 cell line showed both higher exosome production and CD151 expression, parallel to *in-vivo* studies in the literature.

## ExoMP-Containing Exosome Detection with the MagLev Sensor Platform

**Figure 6:**
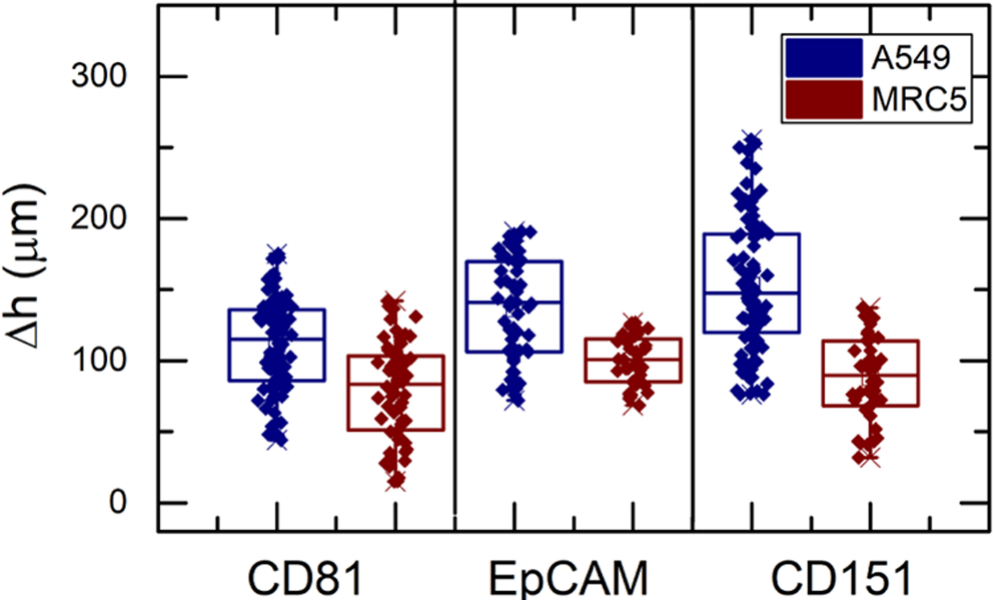
CD81-, EpCAM-, and CD151-containing exosome detection via the MagLev sensor platform; antibody-functionalized PSMs were used against exosomes with various dilution factors (*N* = 40).

Detection of exosomes that were isolated from cell culture supernatants was carried out via MagLev. PSMs functionalized with anti-ExoMP antibodies were used to assess the capability of the MagLev platform for *in-vitro* exosome detection. Exosome isolates were introduced into single Ab-functionalized PSM suspensions. MagLev height measurement was carried out after incubation as previously described. In Figure 6, each data point resembles a single PSM, possibly interacting with several exosomes. Each experiment was run in triplicates, and at least thirty PSMs were visualized in each run.

Figure 6 depicts the results obtained when functionalized PSMs were used as capturing agents. All data collected from the experiment showed a normal distribution, and ANOVA that was performed revealed statistically significant differences between the populations in terms of their means (*p* < 0.05). As shown in the figure, A549 had higher expression of CD81, which was in correlation with immunostaining results. Also, as described in the literature, cancerous cells generate a greater number of exosomes compared to healthy ones, as encountered in this study.^16^ EpCAM detection in samples was also concluded in similar results, with A549 having a greater Δ*h* value, indicating a higher number of EpCAM-containing exosome secretion in cancerous cell lines.

Literature reported that blood samples obtained from NSCLC patients showed a significantly higher level of CD151 expression in their exosomes.^13,14^ Hence, CD151 was targeted as a cancer biomarker in this study. A higher Δ*h* value was obtained from A549 cell line-derived exosomes, whereas no significant Δ*h* difference was encountered when samples isolated from MRC5 were analyzed. The acquired outcome was in parallel with clinical studies in the literature; a greater amount of CD151 was observed in exosomes generated by the NSCLC cell line A549 compared to the non-cancerous cell line; this was also validated via immunostaining. Overall, the sensor platform was proved to be able to distinguish exosomes sourced from healthy cell lines and cancerous cell lines. Compared to current golden standards, such as ELISA or flow cytometry, which requires complex equipment, expertise, and labeling, MagLev provides ease of use, rapid result generation, low initial investment, and label-free application.

## Conclusions

Herein, a MagLev-based biosensor platform for exosome detection was proposed, and the platform was tested with solubilized ExoMPs, EpCAM as a cancer biomarker, CD151 as an NSCLC biomarker, and CD81 as an exosomal biomarker. Density-based detection and quantification of target proteins were carried out with the developed platform; a dynamic range of 1–100 nM was observed with LoD values of 1.324, 0.638, and 0.722 nM for EpCAM, CD81, and CD151, respectively. The exosomes isolated from the NSCLC type cell line A549 were used as model exosomes for further testing of the MagLev platform. MRC5 healthy lung fibroblast cell line was utilized as a control group. Prior to testing the MagLev platform, exosomes isolated from cell lines were characterized by DLS and immunostaining of ExoMPs. After validating that the isolates contain target ExoMPs and exosomes, the detection capability of the MagLev platform was investigated using *in vitro* exosomes. It was shown that detection of these exosomes that contain target ExoMPs was possible. Moreover, the capability of the MagLev platform to distinguish non-cancerous exosomes from cancerous ones by targeting CD151 as a biomarker for lung cancer was demonstrated. It was revealed that exosomes isolated from A549 showed a higher Δ*h* compared to the healthy cell line, indicating elevated expression levels of CD151 and CD81 ExoMPs. Overall, the MagLev platform has shown the potential to detect exosomes that contain specific ExoMPs as non-cancerous and cancerous biomarkers, specifically for NSCLC. It proposes the unique benefits of fast response time and high sensitivity without the necessity of complex equipment or experienced specialists as opposed to current gold standards. Moreover, being a liquid-based system, compared to methods such as tissue biopsy, it turns out as a much less invasive methodology, which could possibly be integrated into casual check-up procedures. Overall, the developed sensor platform provided visual real-time output, sensitive density-based detection, and a low sample size requirement for simple, rapid, and cost-effective detection of cancer biomarkers.

## Supporting Information Available

Details on descriptive statistics and normality analyses results for the antibody saturation study; also, details of sensory characteristic determination for each of the three ExoMPs’ detection study (PDF)
