# Supplementary material for: Utilizing Magnetic Levitation to Detect Lung Cancer-Associated Exosomes
Source: ACS Sens. 2024 Mar 23;9(4):2043–9. doi: 10.1021/acssensors.4c00011 (PMC11059084; doi:10.1021/acssensors.4c00011)
Supplement: Supplementary file 1 — se4c00011_si_001.pdf [file se4c00011_si_001.pdf]

Utilizing Magnetic Levitation to Detect Lung Cancer-Associated Exosomes

Alper Baran Sözmen, Ahu Arslan-Yildiz*

* Corresponding Author
arslanahu@gmail.com

Izmir Institute of Technology, Bioengineering Dept. Izmir/Turkey

Table 1S. Descriptive statistics and Kolmogoroc-Smirnov normality analyses results of antibody saturation study.

Antibody Concentration (µg/ml)	Mean Mg-Lev Height (µm)	Std. Deviation	P-Value
Control	522.536	33.863	0.789
0.005	505.302	32.969	0.353
0.05	468.442	38.915	0.376
0.25	439.716	32.669	1.000
0.5	409.049	32.337	0.488
1	397.233	32.293	0.616
2.5	390.357	29.520	0.588
5	383.650	29.433	0.463
25	382.385	28.101	0.567

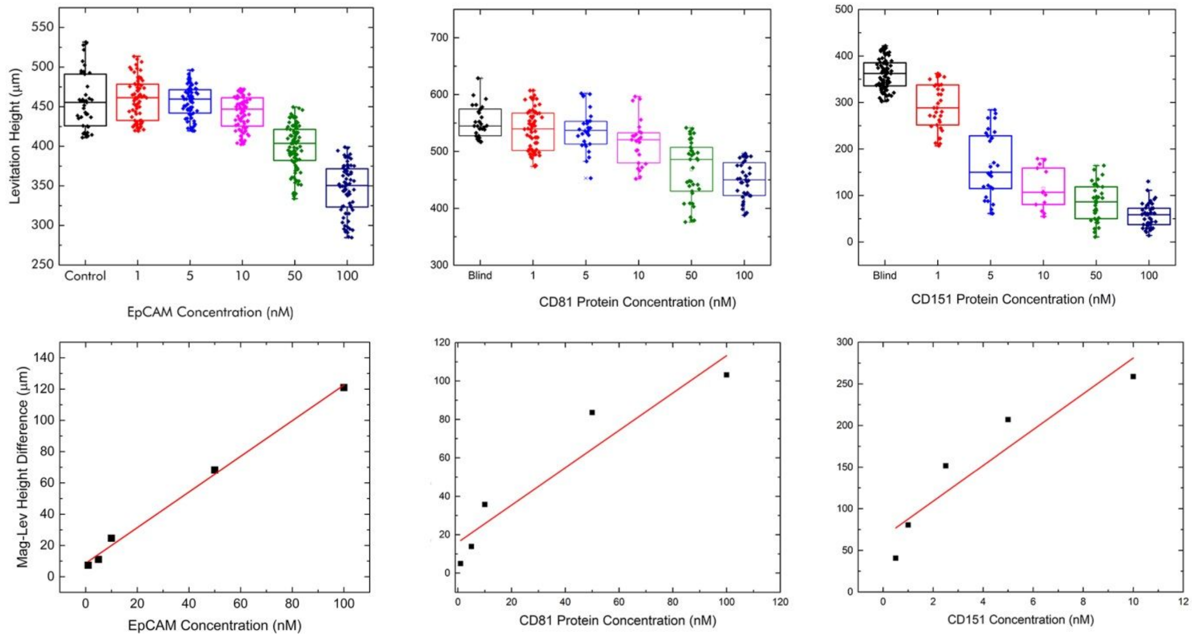

Figure 1S. Protein detection via MagLev sensor platform, whole data of each protein trial (EpCAM, CD81, and CD151) and relevant calibration curves, respectively.
